# Physical mapping of immune-related genes in Yesso scallop (*Patinopecten yessoensis*) using fluorescent *in situ* hybridization

**DOI:** 10.3897/CompCytogen.v10i4.10047

**Published:** 2016-10-25

**Authors:** Zujing Yang, Xuan Li, Huan Liao, Liping Hu, Zhengrui Zhang, Bosong Zhao, Xiaoting Huang, Zhenmin Bao

**Affiliations:** 1 Key Laboratory of Marine Genetics and Breeding (Ocean University of China), Ministry of Education, Qingdao 266003, China; 2 Yantai Fisheries Research Institute, Yantai 264003, China; 3 School of Life Sciences and Biotechnology, Shanghai Jiao Tong University, Shanghai 200240, China

**Keywords:** Cytogenetic map, immune-related genes, FISH, fosmid

## Abstract

The innate immune system plays a pivotal role in defending invasion of microorganisms for scallops. Previous studies on immune-related genes in the Yesso scallop, *Patinopecten yessoensis* (Jay, 1857) have mainly focused on characterization and expression pattern in response to bacterial challenge, no research has been carried out on the cytogenetic level yet. In the present study, eight fosmid clones containing the sequences of key immune-related genes (*Py*NFkB, *Py*TRAF2, *Py*TRAF4, *Py*TRAF7, *Py*Myd88-1, *Py*Myd88-3, *Py*MKK-7 and *Py*TNFR) were isolated and seven of them were successfully mapped on chromosomes of *Patinopecten yessoensis* utilizing fluorescence *in situ* hybridization. Wherein, *Py*Myd88-1, *Py*Myd88-3 and *Py*MKK-7 located on the same chromosome pair with adjacent positions and the other genes were mapped on four non-homologous chromosome pairs, showing a similar distribution to another five model species. The isolation and mapping of such genes of the Yesso scallop will lay a foundation for studies such as assignment of interested genes to chromosomes, construction cytogenetic maps and so on.

## Introduction

Studying chromosomal distribution pattern of important functional genes at the cytogenetic level, which can be useful for discussion of gene evolution, chromosome rearrangement and so on, is a crucial part of genome research in any species. For example, by mapping Antennapedia (ANTP) class homeobox genes to the chromosome in a lophotrochozoan protostome, Hui et al. (2012) elucidated an aspect of the genome organization of the protostome–deuterostome ancestor and revealed high levels of macrosynteny between *Platynereis dumerilii* (Audouin & Milne-Edwards, 1834) and chordates. In scallops, through comparing chromosome location of histone H3 genes in four species, it was suggested that gene duplication/diminution as well as chromosome rearrangements by inversion and translocation may have played important roles in the genomic evolution of Pectinidae (Zhang et al. 2007).

However, limited researches on chromosomal localization of functional genes have been conducted in mollusk species as yet. This is mostly due to the short probe length and the technical limitations, genes and sequences with low copy numbers tend to be more difficult to obtain positive signals after fluorescence *in situ* hybridization (FISH) (Wang et al. 2005, Zhao et al. 2015). To tackle this obstacle, large insert clones were introduced to FISH mapping and used as the probes. Bacterial artificial chromosome (BAC), bacteriophage P1 and fosmid have been already successfully applied in bivalve to achieve the goal of chromosome mapping (Wang et al. 2005, Zhang et al. 2008, Feng et al. 2014). What is more, in the Zhikong scallop, *Chlamys farreri* (Jones & Preston, 1904), Toll-Like receptor signaling pathway genes were unambiguously mapped to the chromosomes utilizing gene-containing BAC clones (Zhao et al. 2015). Such studies brought a novel method to investigating chromosomal distribution pattern of functional genes in mollusk.

The Yesso scallop, *Patinopecten yessoensis* (Jay, 1857), is a cold water bivalve and is naturally distributed along the coastline of northern Japan, the Far East of Russia and the northern Korean Peninsula (Shumway 1991). It is a species of great economic importance in China and Japan. Over the last decade, the population of the Yesso scallop is decreased sharply due to various infections. Like other scallop species, *Patinopecten yessoensis* lacks adaptive immunity and has evolved a series of sophisticated strategies to recognize and eliminate various invaders (Song et al. 2015). Several studies about immune-related genes in the Yesso scallop were published during 2015 to 2016 (Li et al. 2015, Ning et al. 2015, Wang et al. 2015, Zhang et al. 2015, Zou et al. 2015, Xing et al. 2016). These researches identified and characterized of *Py*MyD88, *Py*NFkB, *Py*TRAF, *Py*MKK, *Py*Tollip and *Py*TNFR genes in *Patinopecten yessoensis* and their distinct expression pattern in response to bacterial challenge. There is no doubt these results provided novel insight into immune-related genes of the Yesso scallop and the specific role and response of certain pathways in host immune responses against different bacterial pathogens. Yet, no early study focused on discussing this type of genes on cytogenetic level and mapping them to the chromosomes.

Cytogenetic studies of the Yesso scallop showed it possessed 38 chromosomes with a karyotype formula of 3m+5sm+8st+3t (Huang et al. 2007b). Yet, most of these studies were mainly focusing on the multi-copy genes or sequences such as rDNA sequences and histones genes (Huang et al. 2007b, Zhang et al. 2007). Recently, our study of mapping tandem repeats using fosmid clones demonstrated such probes provide a high successful rate for FISH (Li et al. 2016). Hence, with genome information of immune-related genes, it is an approachable way to locate genes of *Patinopecten yessoensis* using the fosmid clones as probes.

In present study, to investigate the chromosome distribution of immune-related genes of the Yesso scallop, we selected 8 gene-containing fosmid clones as probes for FISH. As a result, we successfully mapped 7 immune-related genes to the chromosomes of the Yesso scallop. Fosmid clones were proved to have high efficiency in chromosome mapping of single or low copy genes in the Yesso scallop. Our results provided the first physical mapping of immune-related genes in *Patinopecten yessoensis*, aiding to understanding chromosomal assignment and evolution of these genes.

## Methods

### Chromosome preparation

Trochophore larvae of *Patinopecten yessoensis* was treated for 2h at room temperature with 0.02% colchicine in sea water, the larvae were exposed to potassium chloride (KCl) solution (0.075M) for 30 min. Then it were fixed three times (15 min each) in fresh Carnoy’s fixative (ethanol:glacial acetic acid = 3:1 v/v). Chromosome spreads were obtained by dissociating fixed larvae in 50% acetic acid and dropping the cellular suspension onto slides heated to 56°C.

### Selection of fosmid clones and probe labeling

A fosmid library including 122, 880 clones of *Patinopecten yessoensis* has been constructed in our lab recently. The restriction fragments of two-dimensionally pooled fosmid clones were sequenced and generated sequences by a physical mapping technology based on next-generation sequencing, whole genome profiling (WGP) (Oeveren et al. 2011). The strategy for pooling clones was that clones in eight 384-microwell plates were mixed for a super pool. And two kinds of type IIB restriction endonucleases named BsaXI and FspEI were used to generate digested tags. These sequence tags are assigned to individual fosmid clones with a decoding rate over 88%. Sequences of 19 immune-related genes were identified by our lab (Li et al. 2015, Ning et al. 2015, Wang et al. 2015, Zou et al. 2015, Xing et al. 2016). These sequences were then cross checked with those sequence tags in order to locate genes to mono-clone.

Plasmid DNA from gene-containing fosmid clones, with an average insert size of 30-45 kb, was extracted by standard laboratory method (Sambrook and Russell David 1989) and labeled with digoxingenin-11-dUTP or biotin-16-dUTP using Dig- or Biotin-Nick Translation Mix (Roche) following the manufacturer’s instruction. Labeling reaction mix with a total volume of 20µl contained 1µg plasmid DNA and 4µl nick-translation mix and was incubate under 15°C for 90 min. After adding EDTA (Ethylenediaminetetraacetic acid) to a final concentration of 25mM and heating to 65°C for 10 min, aliquots of 2µl reaction mix were sampled and run on 1% agarose gels to monitor fragment size. The length of the probes should be between 100 bp and 600 bp. Labeled probes were purified by SanPrep PCR products purify kit (Sangon Biotech) and then resolved at a concentration of 5-10 ng/µl in a hybridization solution of 2×SSC (sodium chloride/sodium citrate), 50% deinoized formamide and 10% dextran sulphate. 18S-28S rDNA probe were obtained by PCR amplification and labeled with biotin-16-dUTP based on previous study (Huang et al. 2007b).

### FISH and Co-hybridization

FISH experiments were performed following methods previously published (Huang et al. 2007b). Chromosomes were denatured in a mixture containing with 70% formamide and 2×SSC at 76°C for 2 min 30 sec, dehydrated with a series of precool ethanol (70%, 90%, 100%; 5 min each) and air-dried. The hybridization mix was denatured at 90°C for 5 min and cooled rapidly. After incubating with the hybridization mix for 16h at 37°C in a moist chamber, slides were washed once in 50% formamide and 2×SSC for 5 min, three times in 2×SSC at 37°C (5 min each). Signal detection was performed using anti-digoxigenin-rhodamine (Roche) and fluorescein avidin DOS (Vector). Slides were counterstained with DAPI (4’, 6-diamidino-2-phenylindole) in antifade solution (Vector). Microscopic analysis and capture of chromosome images were carried out using a Leica DM4000B microscope equipped with an epifluorescence system and the appropriate filter sets for fluorescein, rhodamine and DAPI as well as CCD camera. The signals were collected and processed with FISH software (Leica CW4000 CytoFISH Version Y 1.3.1). Karyotype analysis was carried out according to criteria defined by Levan et al. (1964).

Co-hybridization was conducted to investigate the relative chromosomal positions between each two fosmid clones. The protocol follows the same procedure of regular hybridization. And the hybridization mix with a total volume of 30µl contained 5-10ng/µl of each probe, 50% formamide, 10% dextran sulphate and 2×SSC.

### Sequencing and sequence analysis

The partial sequences of genes which were successfully located by FISH were amplified and sequenced to make sure those are gene-containing fosmid clones. Primers were published previously (Li et al. 2015, Ning et al. 2015, Wang et al. 2015, Zhao et al. 2015, Xing et al. 2016). Because the total sequence length of *Py*TRAF4 gene are over 40k, the plasmid of fosmid clone did not cover the entire *Py*TRAF4 sequence so that early published primers of this gene were not applicable for present study. Thus, primers of *Py*TRAF4 were redesigned using Primer5 software (Lalitha 2000). The amplification mixture contained 50ng genomic DNA, 0.2µM of each primers, 0.2µM of dNTP, 2mM MgSO_4_ and 0.5U Platinum Taq DNA Polymerase. Cycling conditions were as follow: 2 min at 94°C (denaturation); 30 cycles of 15s at 94°C, 30s at 60°C, and 1min/kb at 68°C for extending. Detailed information about the primers can be found in Table 2. The products were purified with SanPrep PCR products purify kit (Sangon Biotech) for double end-DNA sequencing by ABI3730. Sequences were subjected to sequence similarity searches using BLASTN.

## Results

### FISH mapping

**Figure 1.**
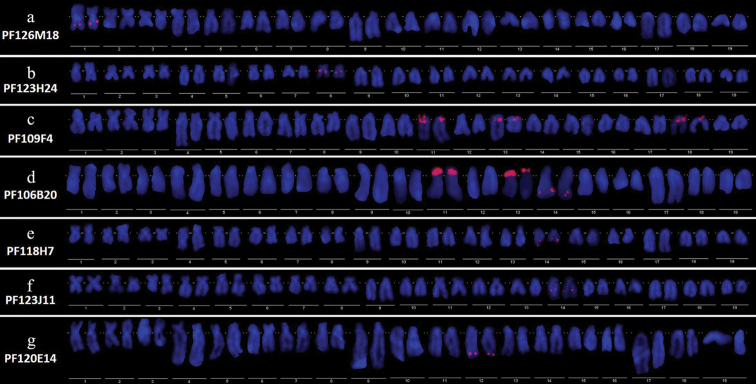
Karyotype analysis results of mapped gene-containing fosmid clones: PF126M18 (**A**); PF123H24 (**B**); PF109F4 (**G**); PF106B20 (**D**); PF118H7 (**E**); PF123J11 (**F**); PF120E14 (**G**). Chromosome numbering is based on chromosome type and relative length. Wherein, chromosome pair 1, 2, 3 represent the metacentric (m) chromosomes. Chromosome pair 4, 5, 6, 7, 8 represent the submetacentric (sm) chromosomes. Chromosome pair 9, 10, 11, 12, 13, 14, 15, 16 represent the subtelocentric (st) chromosomes. Chromosome pair 17, 18, 19 represent the telocentric (t) chromosomes.

In total 19 genes were used for fosmid clones searching. Eight of them were managed to find matched fosmid clones (Table 1). Positive and stable FISH signals were observed in at least 25 analyzed metaphases for each probe. After FISH, 7 of the 8 fosmid clones were successfully mapped to chromosomes of the Yesso scallop (Table 1). Wherein, Clone PF126M18 was mapped to a single metacentric chromosome pair 1 on interstitial positions. (Fig. 1a). Clone PF123H24 was mapped to a single submetacentric chromosome pair 8 on interstitial positions (Fig. 1b). Interestingly, both clone PF109F4 and PF106B20 showed its loci on 3 chromosomes pairs and both have two pairs of loci located on the telomeric position of subtelocentric chromosome pair 11, 13 (Fig. 1c, d). However, the third pair of loci of PF109F4 were found on the telocentric chromosome pair 18, that of PF106B20 were proven to be located on the subtelocentric chromosome pair 14. Clone PF118H7, PF123J11 and PF120E14 were respectively located on a single subtelocentric chromosome pair (Fig. 1e, f, g).

**Table 1.** Information of genes and fosmid clones and FISH mapping results.

Clone name	Gene name	Chromosome type*	Location of signals
PF118H7	*Py*MKK7	st	On telomeric region of 14q
PF123J11	PyMyd88-1	st	On middle region of 14q
PF106B20	PyMyd88-3	st	On telomeric region of 11p, 13p and 14q
PF123H24	PyTRAF7	sm	On centromeric region of 8q
PF109F4	PyNFkB	t	On telomeric region of 11p and 13p, centromeric region of 18q
PF120E14	*Py*TRAF4	st	On telomeric region of 12q
PF126M18	PyTNFR	m	On middle region of 1q
PF118E11	*Py*TRAF2	N/A	No positive signals were found

* m : metacentric , sm : submetacentric , st : subtelocentric , t : telocentric

**Figure 2.**
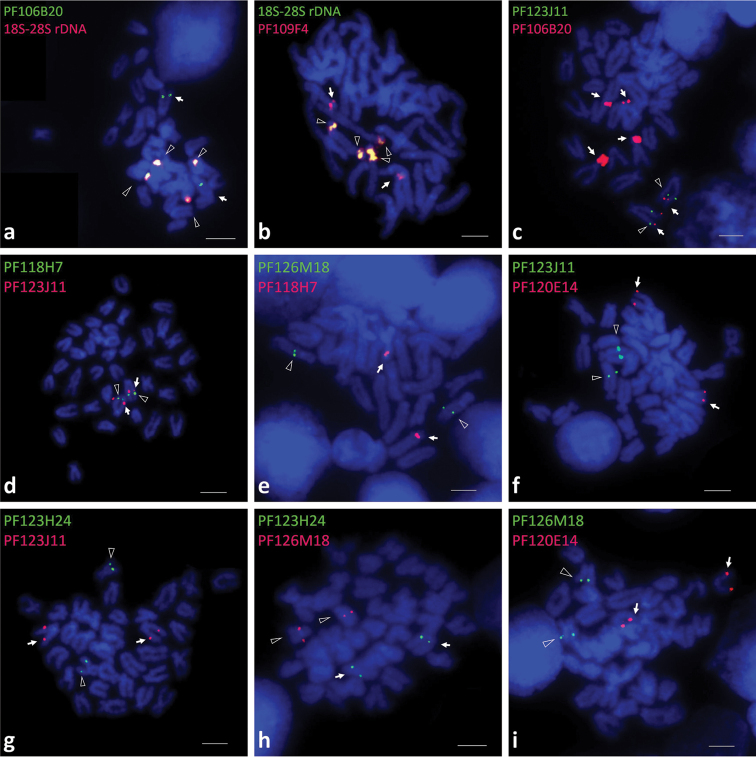
Co-hybridization results of fosmid clones with positive signals on mitotic metaphase chromosomes of *Patinopecten yessoensis* (**A-I**): Mapping of clone PF106B20 & 18S-28S rDNA (**A**), clone PF109F4 &18S-28S rDNA (**B**), clones PF123J11 & PF106B20 (**C**), clones PF118H7 & PF123J11 (**D**), clones PF126M18 & PF118H7 (**E**), clones PF123J11 & PF120E14 (**F**), clones PF123H24 & PF123J11 (**G**), clones PF123H24 & PF126M18 (**H**), clones PF126M18 & PF120E14 (**I**). The arrows and the open triangles indicate positive signals of different probes. Bars=10 µm.

In previous study, clusters of 18S-28S rDNA were localized on 2 different pairs of subtelocentric chromosomes of the Yesso scallop (Huang et al. 2007a). We found some of the mapping results of clone PF106B20 and PF109F4 were highly similar with that of the 18S-28S rDNA, therefore, these two fosmid clones were separately co-hybridized with 18S-28S rDNA. As in Fig. 2a, b, the positions of signals of rDNA obviously matched two pairs of signals of PF106B20 or PF109F4 and the color of red blended with green emerging a yellow look. This suggested that the sequences of clone PF106B20 or PF109F4 were partially similar with 18S-28S rDNA sequences and the extra signals represented the chromosome loci of gene *Py*Myd88-3 and *Py*NFkB.

In order to find out the chromosome position relations of the genes which were successfully mapped, karyotype analysis of FISH results were carried out firstly (Fig. 1). The results showed clones PF126M18, PF123H24 and PF109F4 were located on a pair of metacentric chromosomes, a pair of submetacentric chromosomes and a pair of telocentric chromosomes respectively (Fig. 1a, b, c). Thus gene *Py*TNFR, *Py*TRAF7 and *Py*NFkB must not cluster with other mapped genes according to the significant morphological differences of those chromosomes.

For those fosmid clones whose position relationship couldn’t justified by karyotype, co-hybridization was conducted (Fig. 2). As it can be seen in Fig. 2c, d, clone PF106B20, PF123J11, PF118H7 were mapped to the long arms of the same pair of subtelocentric chromosomes which indicated *Py*Myd88-3, *Py*TRAF7, *Py*MKK7 belonged to the same chromosome pair. Moreover, the co-hybridization results showed the positive signals of clone PF126M18 (*Py*TNFR), PF120E14 (*Py*TRAF4) and PF123H24 (*Py*TRAF7) all distributed in the different chromosome pair with clone PF123J11 or PF118H7 (Fig. 2e, f, g). Clone PF126M18 was also separately proven to be located in different chromosome pair with clone PF123H24 and PF120E14 (Fig. 2h, i). As a result, besides of clone PF106B20, PF123J11, PF118H7, no other clones were found to be mapped to the same chromosomes.

### Sequencing results

The partial sequences of genes from the 7 mapped fosmid clones were amplified and products were successfully sequenced. A BLASTN analysis of the 7 sequences against the *Patinopecten yessoensis* genome sequencing data showed significant sequence matches as we expected and confirmed the existence of the genes in the fosmid clones (Table 2).

**Table 2.** Primers used for PCR and BNALSN results of the mapped clones.

Gene name (Clone name)	Primer	Primer sequence(5’-3’)	Identities
*Py*Myd88-1 (PF123J11)	F- PF123J11 R- PF123J11	TTGCACATGCTCTGTCGCC GGACGCAGTTCGCTTTTGAT	679/684 (99%)
PyMyd88-3 (PF106B20)	F- PF106B20 R- PF106B20	GAGTGTCGAGTGCGACTTCATG CGCCTTCAGTAGACGTTTCCAG	944/946 (99%)
*Py*TRAF7 (PF123H24)	F- PF123H24 R- PF123H24	CCAGATTGTCACGCTGAAAGG CCAGATTGTCACGCTGAAAGG	76/77 (99%)
*Py*MKK7 (PF118H7)	F- PF118H7 R- PF118H7	TCAAAGGCTAAGACGAGGAGTGC CAACCAATGTGATGCCCAGG	773/779 (99%)
PyNFkB (PF109F4)	F- PF109F4 R- PF109F4	TGCCCGTGTTGTGGTAACCTTGG CGTGAGAGAGTTTTGTCCGCCCTT	90/92 (98%)
PyTNFR (PF126M18)	F- PF126M18 R- PF126M18	AACAACCTACCTGAAACGGAACA CGGTTAGGATTTGGACAAGGAC	54/56 (96%)
PyTRAF4 (PF120E14)	F- PF120E14 R- PF120E14	GGACTTATCGCTGTCAACC AAATGTGGCACCTTACTCG	187/189 (99%)

Particularly, because of the similarity between mapping results of clone PF106B20 and 18S-28S rDNA, PF109F4 and 18S-28S rDNA, plasmids of these two clones were used as template for amplification of 18S-28S rDNA. The obtained PCR products were successfully sequenced and the sequences were identical to the 18S-28S rDNA sequence of the Yesso scallop.

## Discussion

The innate immune system plays a vital role in scallop species defending against various invaders because of lacking of adaptive immunity (Song et al. 2015). Focusing on studying such subject would not only provide us insights of molecular mechanism of the strategies that scallop species take for immune defense, but also play a guiding role in scallop farming industry. In 2015, a series of studies which genome-widely identified and characterized immune-related genes in the Yesso scallop were published (Li et al. 2015, Ning et al. 2015, Wang et al. 2015, Zhang et al. 2015, Zou et al. 2015). There is no doubt these studies provide insights into the versatile roles and responses of genes like TRAF genes, Myd88 genes, MKK genes and so on in the innate immune system against Gram-negative bacterial pathogens in bivalves. In addition, by conducting phylogenetic analyses, such studies also investigated evolutionary relationships of immune-related genes of *Patinopecten yessoensis*. Compared with genes from other invertebrate and vertebrate species, it was shown that most of immune-related genes of the Yesso scallop like *Py*Tollip, *Py*NFkB, *Py*MKKs, *Py*TRAFs and so on were found to have relatively conserved structural features. Indubitably, the phylogenetic tree provided valuable information such as orthologous and paralogous relations among genes. At the same time, investigation of chromosomal distribution pattern of genes is another approach to gain a better understanding of gene evolutionary issue.

In present study, *Py*NFkB, *Py*TRAF4, *Py*TRAF7, *Py*Myd88-1, *Py*Myd88-3, *Py*MKK-7 and *Py*TNFR genes were successfully mapped to chromosomes of the Yesso scallop. After co-hybridization, *Py*MKK-7, *Py*Myd88-1 and *Py*Myd88-3 were found to be located on the same chromosome pair with very adjacent positions. Previous studies have shown that genes with similar expression patterns tend to cluster more frequently than those with different expression patterns (Chen et al. 2010, Liu and Han 2009). And looking back to the relative expression level of *Py*MKK-7, *Py*Myd88-1 and *Py*Myd88-3 genes after Gram-negative (*Vibrio anguillarum*) bacterial challenge, it was obvious that the expression levels of all these three genes went through the pattern of first dropping and then increasing (Ning et al. 2015, Zou et al. 2015). Combining the adjacent chromosomal location and expression patterns of these three genes, we proved the hypothesis that the genes clustered on short distance along chromosomal may have similar expression patterns.

The genome-wide identification of five *Py*MyD88 duplication genes in the Yesso scallop showed that all five genes were located on the same linkage group. In present study, fosmid clones of two *Py*MyD88 genes (*Py*Myd88-1 and *Py*Myd88-3) were pinpointed and were successfully mapped to the same chromosome pair of the Yesso scallop. What is more, *Py*MKK7 gene was found to locate on the adjacent chromosomal position with that of *Py*MyD88 genes. It was thought that the higher number of *Py*MyD88 duplications in the Yesso scallop may be relevant to their specific and cooperative functions in the corresponding innate immune system and in *Py*MyD88-dependent and independent pathways against bacterial infection (Ning et al. 2015). With the present outcome, it seems *Py*MKK7 gene may also have cooperative function with *Py*MyD88 genes in the Yesso scallop.

According to genome sequencing data (BioProject number PRJNA259405), *Py*MKK-7, *Py*Myd88-1 and *Py*Myd88-3 genes were contained in three different scaffold, which were scaffold7441 (*Py*Myd88-1), scaffold5789 (*Py*Myd88-3), scaffold11045 (*Py*MKK-7) respectively. Based on the established SNP linkage map (unpublished), scaffold7441 and scaffold5789 belonged to the linkage group 1. However, which linkage group was scaffold11045 distributed to was not clear. Through co-hybridization, the present study successfully mapped *Py*MKK-7 and *Py*Myd88-1 genes into the same chromosome pair and anchored scaffold11045 to the linkage group 1 just like scaffold7441 and scaffold5789 which helped genome assembly of the *Patinopecten yessoensis*.

Apart from the three genes mapped in the same chromosome pair mentioned above, no other genes were found to have the similar pattern in the present study. In the Zhikong scallop, five key TLR signaling pathway genes (*Cf*TLR, *Cf*Myd88, *Cf*TRAF6, *Cf*NFkB and *Cf*IkB) were mapped in five non-homologous chromosome pairs which was similar with the FISH results of *Py*TRAF7, *Py*NFkB, *Py*TRAF4 and *Py*TNFR (Zhao et al. 2015). What’s more, for comparison purpose, we obtained the distribution information of same immune-related genes in five model species (Table 3) through the NCBI database (NCBI Map Viewer, http://www.ncbi.nlm.nih.gov/mapview/). In four species, all the known NFkB, Myd88, MKK, TNFR and TRAF genes locate on the non-homologous chromosome pairs. Only in *Drosophila melanogaster* Meigen, 1830, there are genes locating on same chromosome pairs, such as gene Dorsal and TRAF4 are on the chromosome 2L and gene MKK7 and TNFR are on the chromosome X. Thus, as it established, the candidate immune genes NFkB, Myd88, MKK, TNFR and TRAF are distantly linked in the not only scallops but also other species. This suggested such phenomenon might be universal among organisms.

**Table 3.** Chromosomal localization of immune-related genes in five model organisms.

Organism	NFkB	Myd88	MKK	TNFR	TRAF4	TRAF7
***Homo sapiens***	NFkB2(4791*) Chr. 10	MyD88 (4615*) Chr. 3	MKK7(5609*) Chr. 19	TNFRSF18(8784*) Chr. 1	TRAF4(9618*) Chr. 17	TRAF7(84231*) Chr. 16
***Drosophila melanogaster***	Dorsal(35047*) Chr. 2L	MyD88(35956*) Chr. 2R	MKK7(32256*) Chr. X	TNFR(32849*) Chr. X	TRAF4(33638 *) Chr. 2L	N/A
***Danio rerio***	NFkB3(425099*) Chr. 7	MyD88(403145*) Chr. 24	MKK7(560913*) Chr. 1	N/A	TRAF4b(404035 *) Chr. 21	TRAF7(563746*) Chr. 3
***Mus musculus***	NFkB3(5970*) Chr. 11	MyD88(17874*) Chr. 9	MKK7(26400*) Chr. 8	TNFRSF1B(21938*) Chr. 4	TRAF4(22032 *) Chr.11	TRAF7(224619*) Chr. 17
***Gallus gallus***	NFkB2(386574*) Chr. 6	MyD88(420420*) Chr. 2	N/A	TNFRSF1B(395083*) Chr. 21	TRAF4(417577*) Chr. 19	TRAF7(416555*) Chr. 14

* Gene ID in NCBI GENE database; Chr. : Chromosome .

Moreover, fosmid clone PF109F4 and PF106B20 were mapped to three different chromosome pairs separately. The FISH mapping results of the two clones both showed two pair positive signals located on two subtelocentric chromosome pairs which were quite alike with the FISH mapping image of the 18S-28S rDNA. With PCR as well as sequencing results, it was proved that these two clones did contain partial 18S-28S rDNA sequence. The average insert fragment of fosmid clones applied within this ranged from 30kb to 45kb. As a result, beside covered the target sequences that needed for FISH mapping, it inevitably included other sequences from the Yesso scallop genome. Early study showed 18S-28S rDNA sequence was highly repetitive and also had quite high hybridization efficiency during FISH. As a result, possessing partial homologous sequences of 18S-28S rDNA was believed to be the reason of appearance of multiple positive signals using clone PF109F4 and PF106B20 as probes. The co-hybridization result of PF109F4 and 18S-28S rDNA, PF106B20 and 18S-28S rDNA also proved these phenomena.

FISH is a powerful tool for chromosomal localization of DNA sequences and have already been used for cytogenetic study in scallop species. Comprehensively speaking, published study were mostly focusing on the multi-copy sequencing mapping such as histone H3 gene , ribosomal genes and satellite DNA (Leitão and Chaves 2008, Huang et al. 2006, 2007b, Zhang et al. 2007, Feng et al. 2014). In Zhikong scallop, five Toll-Like receptor signaling pathway genes were mapped into five non-homologous chromosomes and hereby, it was considered that the co-expression of TLR signaling pathway genes in the *Chlamys farreri* may not act in a distance-dependent way (Zhao et al. 2015).

The present study revealed chromosomal distribution pattern of seven immune-related genes and enriched the number of chromosome markers in the Yesso scallop. These results will lay a foundation for the upcoming genome and cytogenetic research in *Patinopecten yessoensis*.
